# Marmoset angiography just by percutaneous puncture of the caudal ventral artery

**DOI:** 10.1371/journal.pone.0250576

**Published:** 2021-04-28

**Authors:** Hiroki Ohta, Teppei Komatsu, Kanako Muta, Makoto Koizumi, Yasuyuki Iguchi, Hirotaka James Okano

**Affiliations:** 1 Division of Regenerative Medicine, Research Center for Medical Sciences, The Jikei University School of Medicine, Tokyo, Japan; 2 Division of Vascular Surgery, Department of Surgery, The Jikei University School of Medicine, Tokyo, Japan; 3 Department of Neurology, The Jikei University School of Medicine, Tokyo, Japan; 4 Laboratory of Animal Facilities, Research Center for Medical Sciences, The Jikei University School of Medicine, Tokyo, Japan; Barrow Neurological Institute, UNITED STATES

## Abstract

Surgery in humans is continuously evolving and promoted minimally invasive treatment. On the other hand, despite the importance of the 3Rs principles for experimental animals is well documented, no reports describe specific methodologies for implementing "refinement" in practice. Here, we describe a new technique, the "Ohta Method" for caudal arthrocentesis in the pursuit of the 3Rs for animal experiments and the development of innovative methods for investigating systemic organ arteries through minimally invasive procedures. This procedure requires only a percutaneous puncture of the caudal artery without any injury to the limb or body trunk. In addition, it does not cut down the artery, making hemostasis easier and recovering arterial damage easier. We will show multiple organ artery angiographies in marmoset for the first time in the world. The principle described in this paper could also be applied to many other small animals, such as rats. Moreover, using this method, multiple doses of the drug or cells can be administered to the target organ at the time of therapeutic intervention, thereby enabling the establishment of more sophisticated and complex therapeutic intervention studies as translational research.

## Introduction

Surgery in humans is continuously evolving, and advances in endoscopic surgery and robotic surgery have promoted minimally invasive treatment. Endovascular treatment techniques also play a significant role in minimally invasive treatment. Many cases in which surgery will not be tolerated because of advanced age or low cardiorespiratory fitness, and operating is therefore judged to be impossible. Endovascular treatment has become safe for such cases [1, 2]. On the other hand, despite the 3Rs principles for using experimental animals [3], no reports describe specific methodologies for implementing “refinement” in practice. We believe that there are several reasons for this gap:

1. Rodents commonly used in the laboratory are much smaller than humans.
2. Sufficiently small laboratory instruments for use in small laboratory animals have not been optimized.
3. Rather sophisticated techniques are required to perform minimally invasive procedures on small laboratory animals.

Animal experiments usually involve a thoracotomy or laparotomy [4] and are highly invasive. Even the most peripheral arterial approach among endovascular approaches that do not manipulate the trunk is limited by manipulating the carotid and femoral arteries. These approach vessels are ligated without reconstruction, and a decline in peripheral blood flow from the approach site cannot be avoided. For example, in a conventional cerebral infarction model, a large incision is made in the neck, and a nylon thread or something similar is inserted through the carotid artery. Then, after the procedure is completed, the carotid artery is ligated [5, 6].

Previous experimental animal models’ design forced intra-arterial approaches from the extremities or trunk at the expense of peripheral arteries [7]. However, in our approach, we do not sacrifice any arteries in the extremities or trunk and are able to perform a minimally invasive transarterial approach. That is, we established a way to approach almost all organs only by puncturing the caudal artery. In addition to this very minimally invasive approach, we aimed to establish a method that enables establishing small laboratory animal models and therapeutic interventions that are equally minimally invasive as those in humans by applying endovascular therapeutic procedures commonly used in humans. To this end, thin wires and microcatheters must be made and, in some cases, custom-made for the use of high-resolution angiography devices to refine the technique. No matter what factors are missing, it will not be successful.

Here, we describe a new technique for caudal ventral arteriocentesis to pursue the 3Rs for laboratory experiments on animals (Replacement, Reduction, and Refinement) through minimally invasive procedures and multiple therapeutic interventions. Because of the ethics of animal experiments, the establishment of a less invasive experimental method is urgent. In addition, developing minimally invasive approaches not only practices the principles of 3Rs in laboratory animals. Multiple doses of the drug or cells can be administered to the target organ at the time of therapeutic intervention, thereby enabling more sophisticated and complex therapeutic intervention studies.

## Materials and methods

### Ethical statement

This study was approved by the Institutional Animal Care and Use Committee of the Jikei University (No. 2015-123C3, 2016–105, 2018–024). All procedures were conducted according to the Fundamental Guidelines for Proper Conduct of Animal Experiments and Related Activities in Academic Research Institutions issued by the Japanese Ministry of Education, Culture, Sports, Science, and Technology.

### Animals

Marmosets acquired from Nihon Clea (CLEA Japan, Inc., Gifu, Japan) or self-propagated on the day of surgery (300~350 g) were used. The marmosets were housed in family cages (W800mm, D650mm, H1500mm), paired cages (W800mm, D650mm, H720mm), or individual/intensive care cages (W400mm, D650mm, H720mm). The room temperature was maintained at 26–30°C. The marmosets were fed 40 g of pellets (LabDiet, PMI Nutrition International LLC, U.S.A.) twice a day supplemented with honey, Vitamin C, and Vitamin D. Environmental enrichment such as wooden toys, climbing structures, and swings were provided depending on the housing conditions. The animal care staff observed the animals’ health and well-being daily using criteria such as fecal condition, appetite, hair condition, and movement. If the staff suspected any problems, they would consult with veterinarians. Each animal was kept in a pain-free state during surgery by appropriately regulating the inhalational anesthetic based on the SpO2 and heart rate. Animals were maintained at a body temperature of 36–38°C during surgery. After the completion of this experiment, they were not sacrificed, but were allowed to survive as breeding stock.

### Animal anaesthesia

Procedures were performed in the hybrid operating room with digital subtraction angiography (Artis Zee, Siemens, Germany) in the Jikei University School of Medicine laboratory animal facilities. Marmosets were anesthetized by injecting 12 mg/kg of alfaxalone into the thigh muscle. After that, a 14-gauge catheter (Terumo, Tokyo, Japan) was visually intubated into the trachea, and controlled breathing was started. Anesthesia was maintained through ventilation using an animal ventilator and 1%-3% isoflurane. The body temperature was maintained using a multipanel heater (Vivaria, Osaka, Japan).

### Caudal Ventral Artery (CVA) puncture

The microcatheter was first flushed with heparinized physiological saline. The marmoset was placed in a supine position with a shaved tail, and the tail skin was sterilized with povidone-iodine. A 22-gauge sheath (Terumo, Tokyo, Japan) was inserted into the CVA. A microcatheter for angiography was inserted into the CVA. After confirmation of arterial blood backflow from the sheath, a wire and microcatheter were inserted as long as no resistance was felt. Upon resistance, the insertion of the microcatheter was stopped immediately, and the position of the microcatheter was confirmed by fluoroscopic imaging.

### Selective cannulation and angiography

The microcatheter was inserted into the abdominal aorta from the CVA. The wire and microcatheter were guided from the abdominal aorta to the center and were raised to the ascending aorta using a digital subtraction angiographic unit (Artis Zee, Siemens, Germany). Only the wire was removed while taking care not to change the position of the microcatheter. The contrast media (Iohexol, Daiichi Sankyo, Tokyo, Japan) at half concentration was rapidly injected from the microcatheter, and arch aortography was carried out, in which 0.15 ml contrast media was injected each time. For subsequent branch cannulation, the contrast images were overlaid for navigation. This procedure can reduce the volume of contrast media needed. We used only approximately 0.1–0.15 ml of half-diluted contrast (approximately 0.2 ml of aortography). The maximum contrast material dose was up to 3 ml.

At the time of cannulation, wire orientation was controlled by slightly bending the wire tip and twisting the hand. The bifurcation position and angle were recognized by aortic angiography described earlier, and the target blood vessel was cannulated. During cannulation of the cervical branch, the C-arm has tilted to view the aortic arch at a right angle. The left internal carotid artery was cannulated, and angiography was performed. For selective cannulation of the superior mesenteric artery (SMA), abdominal aortography was performed at the level 3–4 vertebral bodies caudal from the diaphragm to confirm the SMA’s location direction bifurcation. The same procedure was carried out in other abdominal visceral branches. If the total amount of contrast agent exceeded 3 ml, the inspection was terminated at that time. The sheath was removed to complete the procedure.

In some cases, the sheath punctured in the tail artery was not removed, and the treatment was carried out so that it could not be removed but rather served as a route for blood collection on the following day. Needless aspiration was avoided, following the goal for "refinement" for the welfare of animals. By injecting embolism material through this procedure after selective organ cannulation was performed as described, a minimally invasive organ ischemia model was produced. The microcatheter tip was positioned at the origin of the abdominal visceral branches. The renal artery (RA) was cannulated selectively based on the previous angiography. After the RA was cannulated, angiography confirmed the tip of the microcatheter again.

## Results

### Percutaneous cannulation of the Caudal Ventral Artery (CVA) *"Ohta Method"*

**Fig 1:**
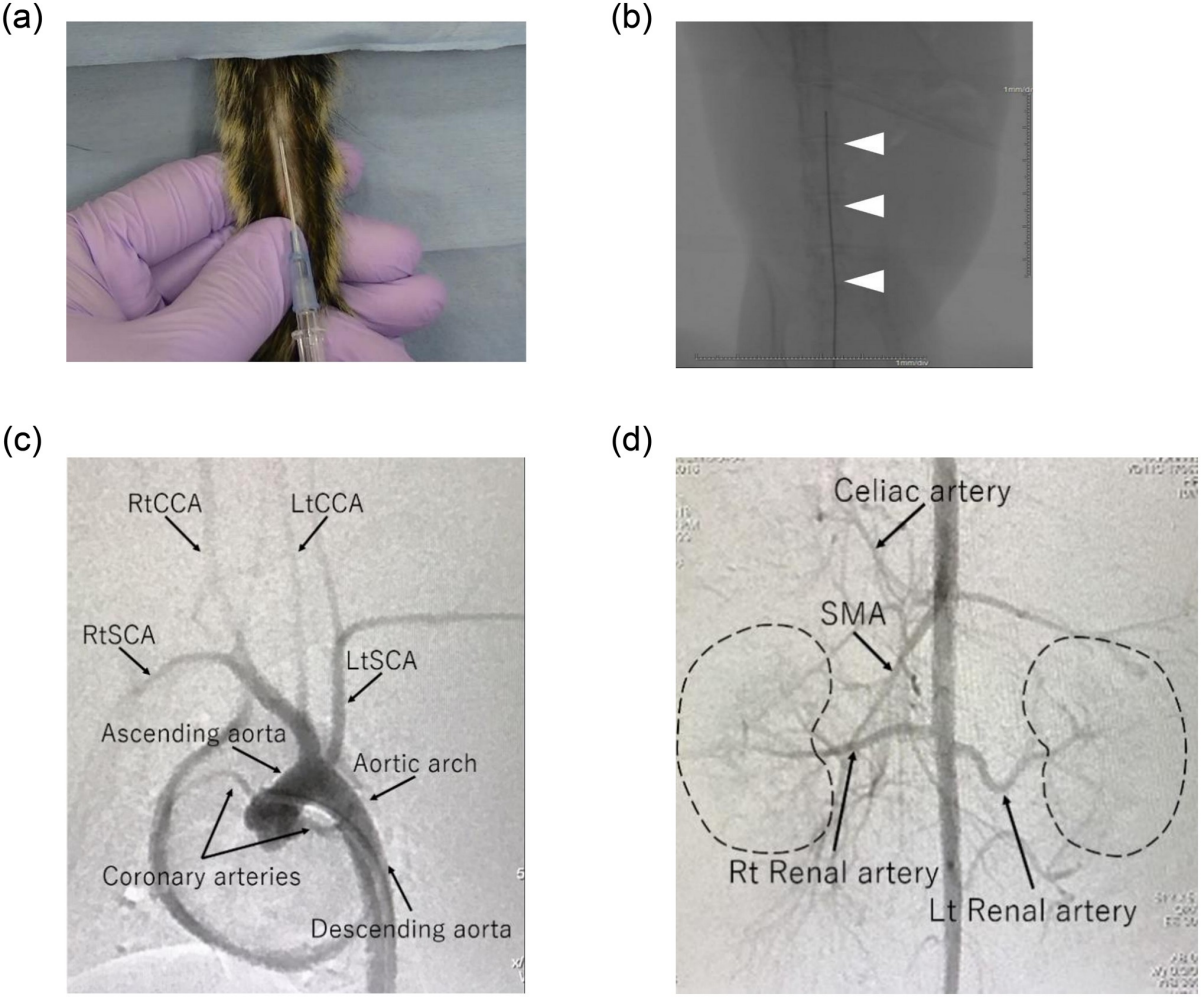
The most minimally invasive aortic approach. (a) Puncture of caudal ventral artery (CVA) in marmosets. A sheath was inserted through the ventral midline, approximately 5 cm from the root of the tail of the marmoset, at a sharp angle after tail shaving. (b) Marmoset fluoroscopic images of the wire inserted carefully through the sheath inserted into the CVA and guided into the abdominal aorta. If the wire is lost in the abdominal visceral branch, it can be withdrawn immediately because it can be confirmed in real time by the fluoroscope. (c,d) A contrast agent was injected into a microcatheter indwelling in the arch/abdominal aorta, and arch/abdominal angiography was performed. There were individual differences in the position and direction of the branch of visceral arteries. Detailed vascular information was obtained in the aortic arch (c) and abdominal aorta (d) of marmosets.

The “Ohta Method” is an ultra-minimally invasive technique in which a 22-gauge sheath (ID: 0.6 mm, OD: 0.9 mm, Terumo, Tokyo, Japan) by percutaneous puncture alone without incising the tail, and a microcatheter or wire is inserted through the sheath and cannulated into vessels of the desired organ using X-ray fluoroscopy. Just by performing percutaneous puncture of the CVA of common marmosets (Callithrix jacchus) was allowed placement of a 22-gauge sheath. This sheath, whose inner tube is a puncture needle, is a highly versatile medical instrument when administering an infusion to humans. The sheath was inserted through the ventral midline, approximately 5 cm from the root of the animal body (Fig 1a), with a sharp angle. As we have already reported [8], when puncturing the CVA, it was necessary to keep the tail and the whole body warm. The sheath could be inserted into the CVA after shaving. We showed these tips as a S1 Fig. Compared with the procedure in the rat, puncturing the CVA in the marmoset was difficult because the skin was hard, and the subcutaneous tissue was loose. Although these data are not presented, the distal approach was also possible after the previous proximal site was punctured using this puncture method. A 0.016" guidewire (Toray Medical Co., Ltd., Tokyo, Japan) was carefully inserted through the sheath inserted into the caudal artery, along which a small-diameter microcatheter (ID: 0.42 mm, OD: 0.55 mm, Kaneko Code Co., Ltd., Tokyo, Japan) could be guided. This wire technique under X-ray fluoroscopy is similar to that performed in humans to safely advance wires through blood vessels (Fig 1b). However, insertion from the caudal artery root into the abdominal aorta was technically challenging and needed much training. It was important that, as the tail artery root is easily spastic, the wire and microcatheter are through there as soon as possible after the insertion. The microcatheters used are thinner than those commercially available and are custom-made. Since the microcatheter is custom-made, it fits the 22-gauge sheath and will not be pushed out by blood flow or other factors. Therefore, there was no need for fixation.

### Small animal angiography

After the wire was slightly advanced from the tip of the microcatheter and guided into the ascending/abdominal aorta, the wire was removed, and arch/abdominal aortography was performed (Fig 1c and 1d). In arch angiography, coronary arteries could also be imaged simultaneously. The amount of half-concentrated contrast medium (Iohexol, Daiichi Sankyo, Tokyo, Japan) consumed for a single aortography was approximately 0.15 ml, and sufficiently detailed vascular information was obtained using digital subtraction angiography equipment (Artis Zee, Siemens, Germany). In these angiographies, the artificial respiration management for controlling respiration was suitable; the endotracheal intubation was indispensable for this purpose.

### Selective organ cannulation

We could cannulate various vessels by endovascular operation using wires selectively and microcatheters advanced from the caudal artery. For cannulation of the cervical branch, the cervical branch’s bifurcation was visualized by tilting the C-arm angle to view the aortic arch at an approximately left anterior oblique (LAO) 20 degrees. Although this procedure is fundamental to endovascular treatment, it has been more challenging to perform in laboratory animals than in humans because of the small vessels and thin microcatheters. Care was always taken not to exert excessive force on the tip of the wire to avoid vascular damage.

#### Middle Carotid Artery (MCA)

**Fig 2:**
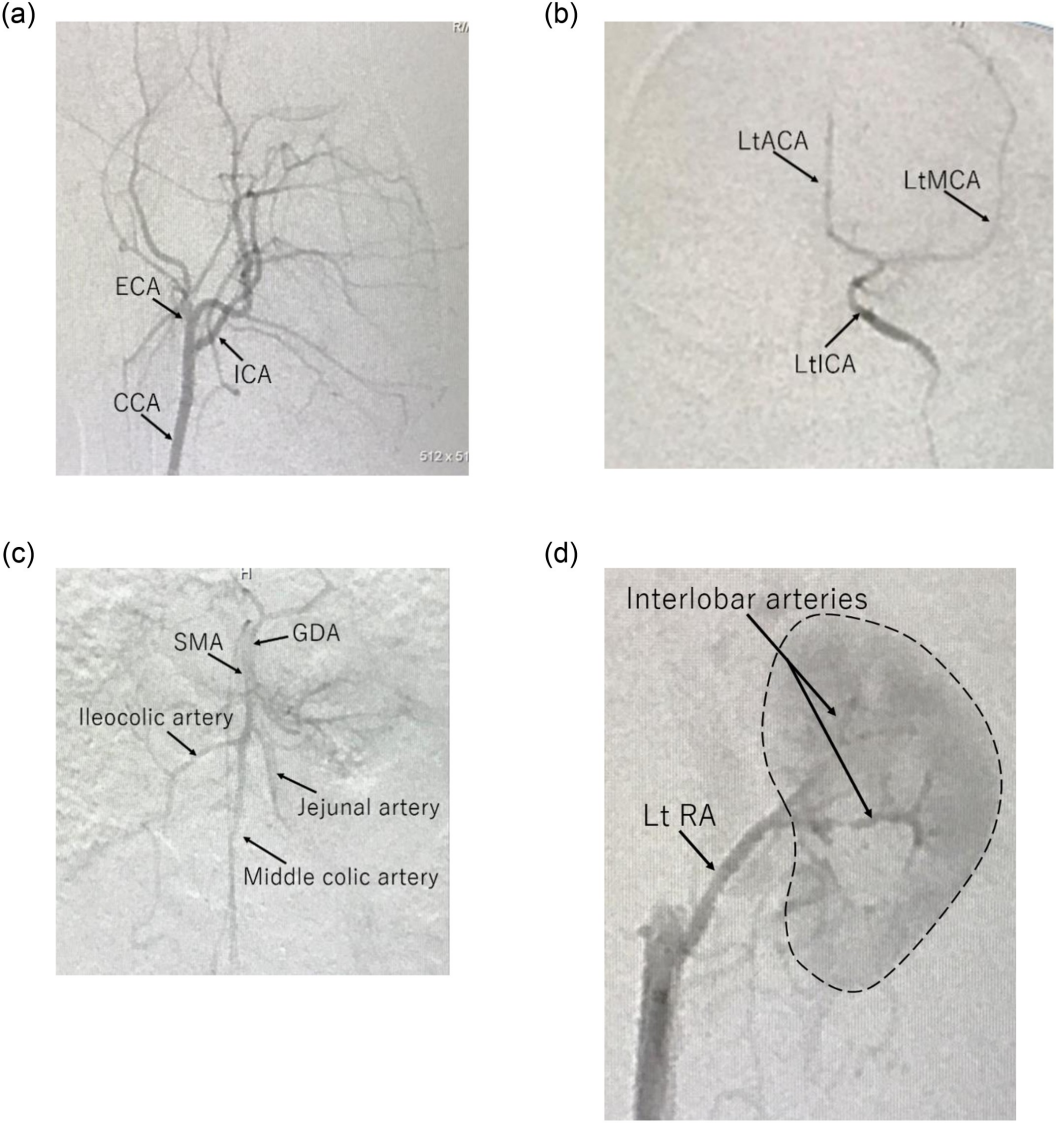
Marmoset angiography. (a) CCA angiography of a marmoset. (b) Angiographic images of selective cannulation into the left internal carotid artery (ICA) with frontal view in the marmoset. (c) The SMA was cannulated with wires and microcatheters, and angiography was performed by injecting a contrast agent through the tip of the microcatheter located at the origin of the SMA in the marmoset. Marmosets showed good visualization of the first bifurcation up to the periphery of the SMA. (d) Wires and microcatheters were cannulated in the RA based on the results of abdominal aortography, as well as the SMA procedure, and selective angiography was performed in the marmoset. Detailed vascular information was obtained up to the interlobar RA. Using a thin microcatheter, more peripheral cannulation and super-selective angiography were successfully performed.

Cerebral angiography was successfully performed by selective cannulation of the left common carotid artery (Fig 2a). The cerebrovascular structures of marmosets are more similar to those of humans than those of rodents. Angiography was performed to confirm the course of the left internal carotid artery (ICA). The selection of the angle at which the bifurcation of the target vessel is well separated was outstanding because the skull’s blood vessel runs three-dimensionally in a complicated pattern. The angle that best demonstrated the course of the ICA in marmosets was LAO 55 to 80 degrees. The wire and microcatheter were raised from the caudal artery to the aortic arch retrogradely, and angiography was carried out. After insertion of the cannula into the left common carotid artery was confirmed, the left ICA was cannulated, and cerebral angiography was carried out (Fig 2b). The angle that best showed the bifurcation of the anterior cerebral artery (ACA) and MCA in marmosets was 0 degrees in front. Insertion of a wire or microcatheter into the ICA periphery should be performed as carefully as possible, and unnecessary stress on the blood vessel wall should not be applied. Small arteries easily spasm, which is troublesome and did not improve much with the use of drugs.

#### Superior Mesenteric Artery (SMA)

The SMA was cannulated by referring to the abdominal aortography image. Then, angiography was performed by injecting a contrast agent through the tip of the microcatheter located at the SMA’s origin (Fig 2c). Although there were rarely problems in the frontal view (LAO 0 degrees) when cannulating the SMA from the abdominal aorta, a tilt of the viewing angle to LAO 80 degrees when the bifurcation was approaching the right renal artery (RA) was sometimes beneficial. Because the SMA is the most critical blood vessel in the visceral branch in both laboratory animals and humans, the cannulation must be carried out carefully. Notably, the wire must not accidentally penetrate the periphery. Researchers need to be careful as the force applied to the vessel wall increases arterial dissection risk.

#### Renal Artery (RA)

Abdominal angiography was also usually performed during selective cannulation of the RAs to determine the position and direction of the bifurcation of the RAs. However, the left RA is often branched almost at right angles and is easy to cannulate without performing abdominal angiography. The RA was the most easily cannulated vessel among the abdominal visceral branches (Fig 2d). However, there are many individual differences in the position and angle of the bifurcation were observed. Abdominal angiography reduced contrast enhancements, which is essential because contrast material load and circulating volume load apply a more significant burden on small animals. Besides, caution should be exercised when blind wire manipulation is performed because if the wire is mistakenly misplaced in the RA, and manipulation is continued without checking the fluoroscopic image, a renal injury may be caused.

## Discussion

Using this technique, which has been applied in the clinic in human, minimally invasive procedures can lead to a “reduction” in suffering and “refinement” of the welfare of laboratory animals, innovative change of veterinary care. In common marmosets, which have attracted attention recently as non-human primates [9], which is an alternative to the rats commonly used in animal experiments, high-resolution imaging equipment and minute intravascular treatment technology can also be utilized in new minimally invasive animal experimental protocols with high safety and high versatility possible. The development of these protocols also helps prevent the high costs of investing in unnecessary clinical trials, laboratory animals’ death, and ineffective drug discovery.

Thin catheters can be inserted into an organ of interest with fluoroscopy and a small amount of contrast material through a method similar to that used in endovascular treatment techniques currently in clinical practice. In both marmosets and rats, when puncturing percutaneously, the CVA runs ventrally in the midline, aiming for the middle to result in a successful puncture. The tail bone of the marmoset projects ventrally around the CVA, making it easy to palpate. However, this procedure requires hard training when performed in small animals. A well-trained researcher can super-selectively place microcatheters to perform imaging and administer substances through the microcatheters. Administering an embolic material creates an animal model of organ ischemia, whereas administering a drug allows drug intervention studies on the models created. Further, the administration of cells would allow cell therapy studies on the model produced. The method described here is a highly versatile method that can be applied in various animal experiments. There is no problem if the needle for blood collection is thin, but the thicker the sheath, the better. There are a wide variety of catheters that can enter the thicker sheath. Therefore, it is also useful in regenerative medicine research, which is currently beginning to show its clinical practice effectiveness [10, 11]. The insertion of a microcatheter through the caudal artery approach allows for selective drug administration and cellular therapies targeting specific organs.

In the previous animal experiments, drug administration and cell therapy have limitations to be performed only once or twice due to technical difficulties. The same caudal approach termed the cut-down method allows only three procedures (maximum) because at least 5–10 mm of avulsion areas are required to expose, cut down the caudal arteries, and hemostasis ligation is essential [12]. With the puncture technique described here, no dissection is required; therefore, a slight shift in the puncture site can be tried, and approaches from the periphery rather than the previous puncture site can be made. Although not shown here, the puncture technique can be employed in one animal 5–6 times. CVA puncture is minimally invasive, and there is no damage to the trunk or extremities. Because this method can be performed just by puncture, it is as invasive as typical intravascular experiments, with only temporary pain. The procedure time is also only a matter of seconds. The cut-down method requires ligation to stop the bleeding, but the puncture method readily achieves hemostasis with compression hemostasis (S1 Table). The major organs of small animals, such as marmosets or rats, can also be cannulated without surgical damage to the extremities. Also, the target organ and the peripheral artery of the organ can be selectively cannulated. Furthermore, securing the CVA route is possible. In this study, we described the method as cannulation to organs, but there are also applications for an arterial blood collection route [13].

The biggest bottlenecks of this method are technical skills and some pitfalls. Experiments should be carried out with a clear understanding of the procedure. We want to refer readers to the S1 Movie shown in the supplementary material regarding proficiencies in the procedure. The animal’s body (including the entire tail) must be kept warm, as we have already reported [8]. Besides, laboratory animals’ unnecessary injury can be avoided by switching to the cut-down technique if puncture fails even after a few trials or by attempting a retry puncture one week later. A contrast medium is essential to guide intravascular microcatheters to the intended site during angiography. Therefore, attention should be paid to allergy development concerns to contrast media in experimental animals and contrast-induced nephropathy. The risk of heart failure due to volume overload and contrast media dose is a critical problem in small animals with low body weight, and efforts should be made to minimize contrast media used in a single administration. With these points in mind, we used only half-diluted contrast.

Moreover, approximately 0.1–0.15 ml of half-diluted contrast (approximately 0.2 ml of aortography) was injected. The maximum contrast media dose was up to 3 ml. The most significant impact on the number of angiographic examinations is radiopaque of microcatheters. Human clinical microcatheters always have radiopaque markers, but similar microcatheters have not been developed for small animals, such as rats or marmosets, and require angiography to locate the microcatheter tip. In addition, because our described technique is very minimally invasive, it can be performed with only local anesthesia in humans but requires general anesthesia in experimental animals in all cases because they are unable to remain at rest and still. Of course, there is also a need for angiography fluoroscopy equipment in laboratory animals, X-ray control areas, and other settings to reduce excess radiation exposure to researchers and laboratory animals. Knowledge of radiation exposure, the angiographic fluoroscopy system itself, and the experimental instruments and devices’ characteristics are also crucial.

This technique is fundamental and revolutionary in animal experiments. We hope that this approach will be carried out by many researchers, leading to a “reduction” in suffering and “refinement” of laboratory animals’ welfare. This technique will also contribute to small animal medicine such as dogs and cats.

## Supporting information

S1 Fig Tips and schema of artery cannulation “Ohta Method”. If performed with these points in mind, it is possible to reach any target organ’s vessels in an ultra-minimally invasive procedure.
(PDF)

S1 Table Comparison of approach methods at the CVA. This table compares the approach methods of the puncture method and the cut-down method. The puncture method is less invasive, less time consuming and is can be performed multiple times. CVA; caudal ventral artery.
(DOCX)

S1 Movie A movie of actual “Ohta Method”. The trick is to do it carefully and slowly until the outer sheath is inserted and then quickly inserted the microcatheter.
(MOV)

S1 File (DOCX)
