# Supplementary material for: Marmoset angiography just by percutaneous puncture of the caudal ventral artery
Source: PLoS One. 2021 Apr 28;16(4):e0250576. doi: 10.1371/journal.pone.0250576 (PMC8081223; doi:10.1371/journal.pone.0250576)
Supplement: S1 Fig — If performed with these points in mind, it is possible to reach any target organ’s vessels in an ultra-minimally invasive procedure. (PDF) [file pone.0250576.s001.pdf]

S1 Fig: Tips and Schema of Artery Cannulation

Tips and Schema of Tail Artery Cannulation *"Ohta method"*

✓ Warm the whole body and tail.
It prevents tail arteries can be spastic. (Ohta H. et al. 2017)

✓ Insert an indwelling needle at the point 5 cm from proximal site of the tail.

✓ Insert an indwelling needle at as acute as possible angle to tail skin surface.
It prevents the indwelling needle piercing the opposite vascular wall.

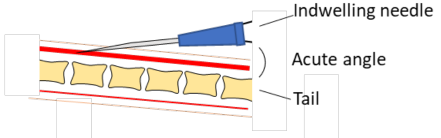

✓ Insert a wire to the indwelling needle as quickly as possible.
It prevents interruption of catheter manipulation.
When a wire or catheter is inserted into a small artery, the strong arterial spasm is easily observed, which can cause the catheter to become stuck and difficult to operate.
