# Supplementary material for: Marmoset angiography just by percutaneous puncture of the caudal ventral artery
Source: PLoS One. 2021 Apr 28;16(4):e0250576. doi: 10.1371/journal.pone.0250576 (PMC8081223; doi:10.1371/journal.pone.0250576)
Supplement: S1 Table — This table compares the approach methods of the puncture method and the cut-down method. The puncture method is less invasive, less time consuming and is can be performed multiple times. CVA; caudal ventral artery. (DOCX) [file pone.0250576.s002.docx]

S1 Table

|  | *Cut down* | *Puncture* |
| --- | --- | --- |
| Surgical technique | Normal | Training required |
| Procedure time | Minimum 10 minutes | Maximum 1 minute |
| Hemostasis | Ligation | Compression |
| Re-do | 2 times | 5 times |
| Agent volume (max) | 3ml | 3ml |
| Invasiveness | Low | Ultra-low |
