# Supplementary figures and images for: Marmoset angiography just by percutaneous puncture of the caudal ventral artery
Source: PLoS One. 2021 Apr 28;16(4):e0250576. doi: 10.1371/journal.pone.0250576 (PMC8081223; doi:10.1371/journal.pone.0250576)

S1 File.


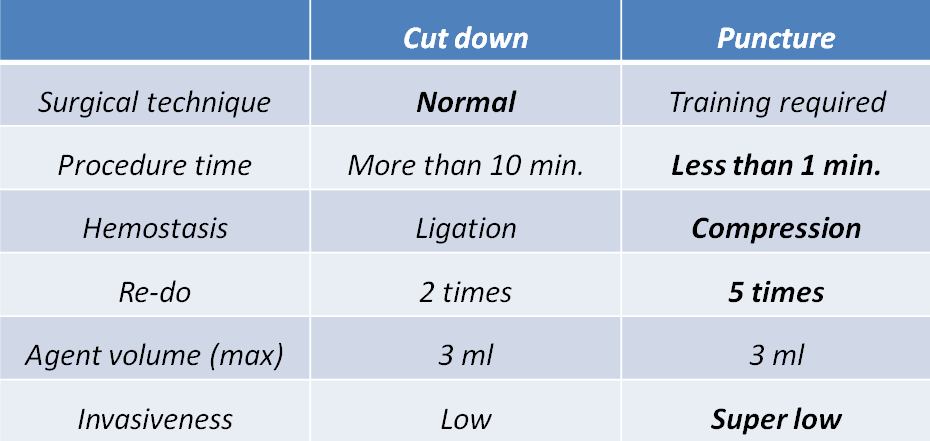

Supplement: S1 File — (DOCX) [file pone.0250576.s004.docx]
